# Exploring the Feasibility of Polysaccharide-Based Mulch Films with Controlled Ammonium and Phosphate Ions Release for Sustainable Agriculture

**DOI:** 10.3390/polym16162298

**Published:** 2024-08-14

**Authors:** Veronica Ciaramitaro, Elena Piacenza, Sara Paliaga, Giuseppe Cavallaro, Luigi Badalucco, Vito Armando Laudicina, Delia Francesca Chillura Martino

**Affiliations:** 1 Department of Biological, Chemical, and Pharmaceutical Sciences and Technology (STEBICEF), Università degli Studi di Palermo, Viale delle Scienze Building 17, 90128 Palermo, Italy; veronicaconcetta.ciaramitaro@unipa.it (V.C.); delia.chilluramartino@unipa.it (D.F.C.M.); 2 Department of Agricultural, Food and Forest Sciences, Università degli Studi di Palermo, Viale delle Scienze Building 4, 90128 Palermo, Italy; sara.paliaga@unipa.it (S.P.); luigi.badalucco@unipa.it (L.B.); 3 Department of Physics and Chemistry-Emilio Segrè, Università degli Studi di Palermo, Viale delle Scienze Building 17, 90128 Palermo, Italy; giuseppe.cavallaro@unipa.it

**Keywords:** polysaccharide-based composite films, water-interaction properties, thermal analysis, mechanical properties, release kinetics, mechanics, mulching

## Abstract

Bio-based polymers are a promising material with which to tackle the use of disposable and non-degradable plastics in agriculture, such as mulching films. However, their poor mechanical properties and the high cost of biomaterials have hindered their widespread application. Hence, in this study, we improved polysaccharide-based films and enriched them with plant nutrients to make them suitable for mulching and fertilizing. Films were produced combining sodium carboxymethyl cellulose (CMC), chitosan (CS), and sodium alginate (SA) at different weight ratios with glycerol and CaCl_2_ as a plasticizer and crosslinker, respectively, and enriched with ammonium phosphate monobasic (NH_4_H_2_PO_4_). A polysaccharide weight ratio of 1:1 generated a film with a more crosslinked structure and a lower expanded network than that featuring the 17:3 ratio, whereas CaCl_2_ increased the films’ water resistance, thermal stability, and strength characteristics, slowing the release rates of NH_4_^+^ and PO_4_^3−^. Thus, composition and crosslinking proved crucial to obtaining promising films for soil mulching.

## 1. Introduction

Over the last 20 years, the global population has explosively grown from 6.0 billion to around 8.0 billion [1], posing threats in terms of food shortages and an increased demand for agricultural products, which has led to the excessive and prolonged use of mulching films based on low-density polyethylene. Once applied to the soil, mulch films increase soil temperatures, reduce water evaporation and fertilizer losses, and help control weed and pest infestations, ultimately increasing crop yields [2]. However, in the medium and long term, these films can cause significant environmental pollution events and side effects on human health [3]. Indeed, due to their low thickness and difficulty in terms of being recovered, they are often abandoned in the soil, intentionally or unintentionally, contributing to farmland meso and microplastic contamination [4]. The growing awareness of sustainable development concepts has prompted researchers to look for innovative, cheap, and alternative mulching films with satisfying properties based on renewable and natural raw materials, such as biopolymers [5]. Biopolymers have frequently attracted the attention of scientists, as these natural materials derive from abundant and renewable sources [6]. According to the American Society for Testing and Materials (ASTM), biodegradable polymers are “the polymers that degrade or decompose under chemical, physical and biological interactions with microorganisms from environments, such as bacteria, fungi and algae” [7]. They decay into natural inorganic (CO_2_ and water) and organic (biomass) components, and do not produce hazardous wastes [8,9]. The demand for biodegradable polymers is ever-increasing because of their properties such as degradability, eco-friendliness, sustainability, and non-toxicity [10]. Among biodegradable polymers, naturally available polysaccharides, such as cellulose, starch, chitin, and alginate, meet all the above requirements, making them suitable candidates for formulations to use as alternative biodegradable plastics [11].

These biopolymers are more expensive than petroleum-based, non-degradable plastics, and their production requires advanced technological processes. Yet, the benefits for nature and a sustainable future could be a forward-looking investment. The polysaccharide mulch films developed to date quite commonly crack and break with use, failing the mandatory requirements. In addition, most films have an excessive degradation rate and poor mechanical, ultraviolet, and water resistance [12], making their large-scale use for agricultural mulching inconvenient. Combining different polysaccharides and adding additives, plasticizers, and crosslinkers enables the modulation of the film’s mechanical, physical, thermal, and chemical properties. Among polysaccharides currently under study, cellulose has widely attracted the interest of researchers due to its properties (e.g., high strength and flexibility, biocompatibility, and non-toxicity), even though its practical use is limited because of its poor water solubility. The latter can be overcome using the water-soluble cellulose-derivative carboxymethyl cellulose (CMC), which also has good film-forming capacity. Composite films that combine CMC with other compounds exhibit superior film performance [13,14]. For instance, adding CMC to a pure chitosan (CS) film improves its tensile strength, thermal stability, water resistance, oxygen permeability, and antibacterial activity [15]. Other studies have demonstrated the synergistic effects of CS and sodium alginate (SA), whereby the latter increases flexibility, and the former improves thermal resistance [16]. SA is widely used to manufacture new materials because of its good moisture absorption, permeability, and high viscosity in aqueous solutions [17,18]. Adding crosslinkers and plasticizers can further improve the stability, flexibility, and toughness of polysaccharide-based films and reduce their brittleness. Crosslinking is a crucial step in optimizing these properties, and several different chemical or physical strategies are available to optimize the crosslinking factors [19,20]. Physical crosslinking relies on the interconnection of non-covalent bonds, such as electrostatic interaction, hydrogen bonding, and the entanglement of molecular chains, among which electrostatic (e.g., ionic and macromolecular) interactions are mostly preferred. In the case of polysaccharides such as SA and CMC, rich in carboxylic groups, calcium chloride (CaCl_2_) is the most widely used crosslinker. Indeed, CaCl_2_ has a low cost and high biocompatibility [21], and Ca^2+^ ions form ionic bonds with -COO^−^ moieties of polysaccharides, bridging their chains and generating a network [21]. Significant factors affecting the ionic bonding between Ca^2+^ and the -COO^−^ of polysaccharides are CaCl_2_ concentration and crosslinking time. Low CaCl_2_ concentrations and short crosslinking times determine inadequate mechanical properties and a loose structure of polysaccharide films, encompassing rapid degradation. On the other hand, increasing CaCl_2_ concentration enables more interactions between Ca^2+^ ions and -COO^−^ groups within films, diminishing their available hydrophilic sites, moisture content, and swelling degree [22], and, in turn, postponing the disintegration of films in water [23]. CaCl_2_ can also improve the tensile strength, elastic modulus, and thermal stability of polysaccharide films [22,24,25]. Yet, drastically increasing the CaCl_2_ concentration or crosslinking time leads to brittle films with low proneness to swell due to their compact structure [26]. Furthermore, -COO^−^ groups within SA and CMC endow polyanionic properties and can also interact with cationic polymers, such as CS. Furthermore, plasticizers can affect the films’ mechanical and physical properties. Plasticizers such as water, glycerol (Gly), sorbitol, or polyethylene glycol increase the free volume between polymer chains and facilitate chain movement [21,27]. For instance, Saravani Pak et al. reported that glycerol could improve the mechanical properties of films, such as tensile strength and elongation, reducing the brittleness of films and increasing the mobility of the polymer chains, in turn promoting film plasticity and flexibility [28]. Some authors have also proven the feasibility of polysaccharide films based on CMC, CS, and SA. For instance, Lan et al. explored the polysaccharide content effect on the properties of the composite film alongside the optimal proportions between CMC, CS, and SA for food packaging applications [29].

Here, we investigated whether polysaccharide-based biodegradable polymer films based on CS, SA, and CMC could also be suitable as mulching films in agriculture. Thus, CMC, CS, and SA were combined at different concentrations and weight ratios compared to previous reports [29], using glycerol as a plasticizer to improve the flexibility and processability of composite films, decrease brittleness, and avoid film shrinkage during handling. To evaluate the reliability of films for mulching, we first focused on studying their mechanical and water barrier performances, brittleness, and sensitivity to moisture. Moreover, the composite films were enriched with ammonium phosphate monobasic (NH_4_H_2_PO_4_) as a new approach to soil fertilization through the release of N and P essential nutrients from the polymeric matrix during film usage and degradation, avoiding the need to encapsulate them as inorganic fertilizers in permeable hydrophobic beads [30,31]. We also evaluated whether adding the crosslinker CaCl_2_ can positively impact the properties of composite films, studying interactions, structural changes, and macroscopic behavior induced by it alongside NH_4_H_2_PO_4_. Finally, the release kinetics of NH_4_^+^ and PO_4_^3−^ ions in water were studied to assess the suitability of these composite films as both mulching and fertilizing substrates for agriculture.

## 2. Materials and Methods

### 2.1. Materials

Chitosan (M_w_ 150–700 kDa, degree of acetylation > 75%, CAS 9012-76-4), sodium alginate (M_w_ 75–150 kDa, CAS 9005-38-3), glycerin (99.5%, CAS 56-81-5), acetic acid (>99.8%, CAS 64-19-2), sodium carboxymethyl cellulose (average M_w_ ~250 kDa, degree of substitution 0.9, CAS 9004-32-4), calcium chloride (>98%), and ammonium phosphate monobasic (CAS 7722-76-1) were purchased from Merk Life Sciences S.r.l. (Milan, Italy). Demineralized water (conductivity < 10 µS/cm) was used in all experiments.

### 2.2. Preparation of Carboxymethyl Cellulose Dispersions, Chitosan-Sodium Alginate Dispersions, and Calcium Chloride Solution

CS/SA and CMC dispersions containing different polysaccharide amounts and ratios were preliminary prepared and explored for obtaining composite films, as reported in the Supplementary Material file (Figures S1 and S2, Table S1). Among these dispersions, those containing 1.5 g of CS, SA, and CMC yielded the most promising and homogeneous results and were subsequently studied in depth.

The dispersion of CMC was prepared by dissolving 1.5 g in 100 mL of distilled water and stirring subsequently with a mechanical homogenizer at room temperature until CMC dissolution.

CS/SA dispersion was prepared by dissolving 1.5 g of CS and 1.5 g of SA in 100 mL of a 2.0% *v*/*v* aqueous acetic acid solution. Then, 2% *w*/*v* glycerol was added to the dispersion and stirred with a mechanical homogenizer at room temperature until SA and CS completely dissolved. Finally, CS/SA dispersion was allowed to stand for 24 h for deaeration.

Then, 2% CaCl_2_ solution was also prepared for the crosslinking of the films.

The resulting CMC or CS/SA dispersions and CaCl_2_ solution were then kept at room temperature until use.

### 2.3. Preparation of Composite Films

First, 1.5% (*w*/*v*) CS/SA and CMC dispersions were mixed using a magnetic stirrer at a CS/SA and CMC weight ratio of 1:1 and 17:3 (i.e., the latter was chosen based on the available literature [29]) and for 30 min. Subsequently, aliquots of 10 g dispersions were cast on Petri dishes, dried in an oven for 24 h at 60 °C, and cooled at room temperature.

Finally, the films were divided into two portions of similar weight (average dried weight of 0.4 g) and appropriately labeled as either the sample or the reference. Reference films were stored; samples were soaked in 2.0% *w*/*v* CaCl_2_ solution for 2 min, retrieved, laid flat on a glass plate, and dried at room temperature.

For comparison, the respective pure films of CS, SA, and CMC were also prepared at the same concentration used for the composite films (1.5% *w*/*v*) and in the same solvents.

From here onward, films will be indicated as CS/SA_CMC 1:1 or 17:3 depending on the weight ratio between the starting dispersions of CS/SA and CMC used for the film’s preparation.

### 2.4. Enrichment of Composite Films with Ammonium Phosphate Monobasic

To assess the potential of the prepared substrates in releasing inorganic fertilizers, films were enriched with 30, 70, or 90% of NH_4_H_2_PO_4_ salt (the salt percentage was calculated based on the average weight of the dry polymer film–i.e., 0.4 g), which was added to the CMC aqueous dispersion. Salt-loaded films were then prepared as described in Section 2.3 using dispersions described in Table 1.

### 2.5. Interaction of Composite Films with Water

The degree of swelling (DS), moisture content (MC), and water-soluble portion of composite films were assessed as described in [32]. Firstly, films were dried in a desiccator containing dried silica gel at room temperature up to a constant weight. The DS in water was determined by immersing four samples of each film of similar weight in distilled water for 1, 2, 3, and 4 h at room temperature. The DC was calculated as follows (1):

Degree of swelling (%) = [(W_a_ − W_b_)/W_b_] × 100, (1)

where W_b_ is the dry weight of the sample and W_a_ is the weight after submersion in distilled water.

The MC was measured by weighing ~0.05 g (dry constant weight) of each film before and after being placed in an oven at 100 °C for 24 h. The MC was calculated by Equation (2):

MC (%) = [(M_0_ − M_f_)/M_0_] × 100, (2)

where M_0_ is the mass of each sample before being placed in the oven and M_f_ is the mass of each sample after being placed in the oven.

Approximately 0.05 g of each film was dried in an oven at 100 °C for 24 h to measure its initial dry mass. Films were then placed in 50 mL of distilled H_2_O at 20 °C for 24 h, and, after the removal of excess water, the wet films were transferred to an oven at 100 °C for 24 h. Their final weight was monitored, and the water-soluble portion percentage was determined using Equation (3):

Water-soluble portion (%) = [(M_0_ − M_f_)/M_0_] × 100, (3)

where M_0_ and M_f_ are each sample’s initial and final weights, respectively.

All experiments were performed in triplicate (*n* = 3) and results are reported as average values with standard deviation (SD).

### 2.6. Thermal and Mechanical Properties of Composite Films

The thermal stabilities, degradation temperatures, and mechanical properties of the films were determined through thermal gravimetric (TGA) and dynamic mechanical (DMA) analyses. For TGA, film portions were processed using a Q5000 IR apparatus (TA Instruments) over a temperature range of 25–400 °C, with a heating rate of 20 °C min^−1^ under a nitrogen gas flow (25 cm^3^ min^−1^), whereas DMA measurements were carried out utilizing a Q800 Dynamic Mechanical Analyzer from TA Instruments (New Castle, DE, USA). The tensile tests were conducted on portions of rectangular-shaped film (10.00 × 5.00 × 0.10 mm^3^), setting a stress ramp of 5 MPa min^−1^ at 25.0 ± 0.5 °C. DMA was performed in triplicate (*n* = 3), and the results are reported as average values with SD.

### 2.7. Attenuated Total Reflection-Fourier Transform Infrared Spectroscopy (ATR-FTIR)

ATR-FTIR spectra were acquired through a Lumos Bruker spectrophotometer (Bruker Instruments, Billerica, MA, USA) equipped with a Platinum ATR unit with a germanium crystal (η = 4.0) operating in the spectral range between 4000 and 600 cm^−1^, a spectral resolution of 2 cm^−1^, and 120 scans. In all spectra, a baseline correction for scattering was made. Data analysis was performed using OPUS 7.5^®^ and OriginPro 2016 software.

### 2.8. X-ray Diffraction (XRD)

The XRD patterns of films were recorded with a Philips PW 1050/39 diffractometer (Philips Analytical, Almelo, The Netherlands). Analyses were performed in the 2-theta range from 5° to 30° using copper Kα radiation (λ = 1.54 Å; setup conditions: tube voltage of 40 kV, current 40 mA, 0.02°/s). XRD patterns and the degree of crystallinity (DC) were analyzed and calculated using Match! version 4 software.

### 2.9. Release Kinetics of PO_4_^3−^ and NH_4_^+^ Ions by Films

Films enriched with NH_4_H_2_PO_4_ salt were immersed in 200 mL of distilled water. Aliquots of 10 mL of solutions were taken after 5, 15, and 30 min, and 1, 4, 8, 24, 48, and 96 h of film incubation, and their absorbance values were recorded using a Shimadzu, UVmini-1240 spectrophotometer (Shimadzu Europe, Duisburg, Germany) at wavelengths of 400 and 667 nm to determine PO_4_^3−^ and NH_4_^+^ concentrations, respectively. The latter were obtained after properly calibrating the instrument with solutions of known PO_4_^3−^ and NH_4_^+^ concentrations. Experiments were performed in triplicate (*n* = 3) and the results are reported as average percentage values (relative to the total amount of salt loaded onto the films) with SD.

Experimental data were subsequently fitted to diverse kinetic models (i.e., zero-order, first-order, second-order kinetics, Higuchi, Hixson-Crowell, and Korsmeyer-Peppas models) [33,34,35] to study the release mechanisms of PO_4_^3−^ and NH_4_^+^ ions from films within the timeframe considered. The best model able to reproduce the experimental trend is a power law known as the Korsmeyer-Peppas model:

Q = M_t_/M_∞_ = kt^n^,

where Q is the amount of analyte released, M_∞_ is the amount of analyte in the equilibrium state, Mt is the amount of analyte released over time t, k is the constant of incorporation of structural modifications and the geometrical characteristics of the system (also considered the release velocity constant), and n is the exponent of release (related to the analyte release mechanism) as a function of time t.

## 3. Results and Discussion

### 3.1. Macroscopic Properties of Polysaccharide-Based Biodegradable Composite Films

Ideally, biodegradable agricultural films should trap and retain water in the soil [36], making it available for plants when necessary, preventing their hydric stress [4,37]; hence, these films must guarantee good water resistance to ensure material structural integrity. In this regard, characterizing the water interaction properties of polysaccharide films is crucial to evaluating their performance in mulching applications, as they give information regarding the material’s ability to hold water in its matrix (degree of swelling), its structural integrity against water excess in rainy conditions (water solubility), and its moisture content, which should be minimized to maintain soil moisture.

**Figure 1:**
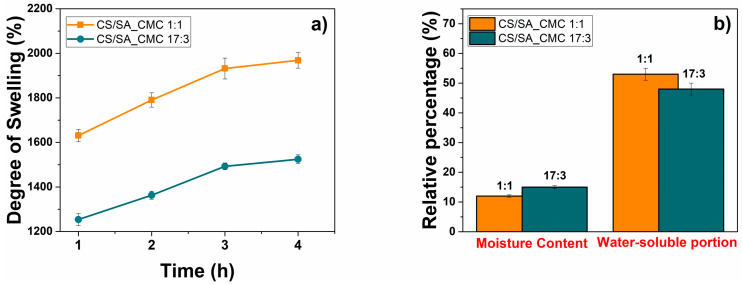
Water-interaction properties of composite polysaccharide films. (**a**) Degree of swelling (%) (**b**) moisture content (%) and water-soluble portion (%) of chitosan/sodium alginate_sodium carboxymethyl cellulose films at a weight ratio 1:1 (orange) or 17:3 (blue).

The degree of swelling (DS), moisture content (MC), and water-soluble portion of the CS/SA_CMC 1:1 and 17:3 films are shown in Figure 1.

Both films show significant DS values and water-soluble portions, aligning with their hydrophilic nature. Indeed, the DS relates to hydrophilic (i.e., carboxylic and hydroxylic) groups in the film structure [38], typical of CS, SA, CMC, and glycerol. Moreover, the polymer chain-glycerol interaction increases the mobility of the matrix, enhancing its affinity with water molecules and making it more susceptible to swelling. In line with DS and water solubility results, the hydrophilic nature of polysaccharides also causes high water vapor molecule adsorption (Figure 1b). Nevertheless, the mass ratio between the polymer components plays a fundamental role in the water interaction properties of films. The CS/SA_CMC 17:3 film displayed a lower DS value and water-soluble portion than the 1:1 counterpart, while an opposite scenario was observed for MC. The former phenomenon may be linked to the formation of a network structure that can reduce the mass loss of composite films in water and hinder their permeation or diffusion when using higher CS/SA amounts. Instead, the 1 (CS/SA): 1 (CMC) weight ratio guarantees greater crosslinking through hydrogen bonds than the 17:3 one, decreasing the free hydroxyl groups of the film matrix available to form hydrogen bonds with water. This event likely leads to a higher ejection of water molecules from the polymeric matrix during solvent casting in CS/SA_CMC 1:1 than in the 17:3 film, which is responsible for the lower MC value in the former. Furthermore, the higher gap between polymeric chains in the 17:3 film than in the 1:1 film facilitates the penetration of water vapor molecules into the polymeric matrix structure, favoring their interaction.

**Figure 2:**
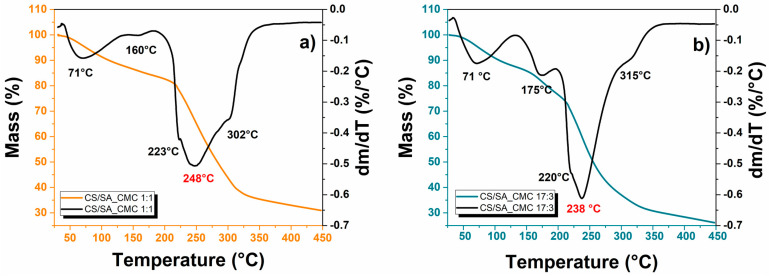
Thermal resistance of composite polysaccharide films. TG curves and DTG curves of chitosan/sodium alginate_sodium carboxymethyl cellulose films in a weight ratio of (**a**) 1:1 or (**b**) 17:3.

The thermal behavior of composite films was investigated by thermogravimetry, and Figure 2 shows the thermogravimetric (TG) and differential thermogravimetric (DTG) curves of CS/SA_CMC films.

Composite films showed three weight loss stages corresponding to the removal of physiosorbed water (25–130 °C), glycerol degradation (130–200 °C), and polysaccharides degradation (200–350 °C) (Table 2).

The first weight loss stage was comparable for both films, whereas the polysaccharide one exhibited a maximum thermal decomposition rate at about 248 °C and 238 °C for the 1:1 and 17:3 films, respectively. These temperatures were intermediate between the ones for the pure polymers of SA (235 °C) [39] and CMC (285 °C) [40], being closer to one of the pure polymers in greater quantity [41]. The shoulder peak at around 220 °C might relate to the partial overlap of different degradation processes (e.g., glycosidic bond breaking, saccharide ring dehydration, or polymer side group decomposition); the shoulder peak at around 300 °C is typical of CS degradation. According to Martino and colleagues, the degradation temperatures of powder, unplasticized, and plasticized CS are 311 °C, 312 °C, and 306 °C, respectively [42]; hence, the peak at 315 °C of the CS/SA_CMC 17:3 film may indicate CS phase separation, likely due to the segregation and re-crystallization of the CS in excess on the surface. In line with this hypothesis, the weight loss and DTG peak referring to glycerol degradation increased with the increment in CS content (Table 2) due to the glycerol portion that did not act as a plasticizer in the 17:3 film, which features a higher evaporation temperature than the plasticized one [43].

Furthermore, this glycerol portion binds water molecules and increases the film’s humidity, corroborating the higher MC for this film than its 1:1 counterpart. Adding a large amount of CS decreased the thermal stability of the film, as highlighted by the lower maximum thermal degradation temperature of the third stage and the residual mass at 450 °C of the 17:3 (238 °C and ca. 26%) film than the 1:1 (248 °C and ca. 31%) film. Similar to the results obtained for the interaction of these films with water, this different behavior may derive from the higher amount of hydrogen bonds formed between CS, SA, and CMC when using the 1:1 ratio rather than the 17:3 weight ratio of CS/SA and CMC.

**Figure 3:**
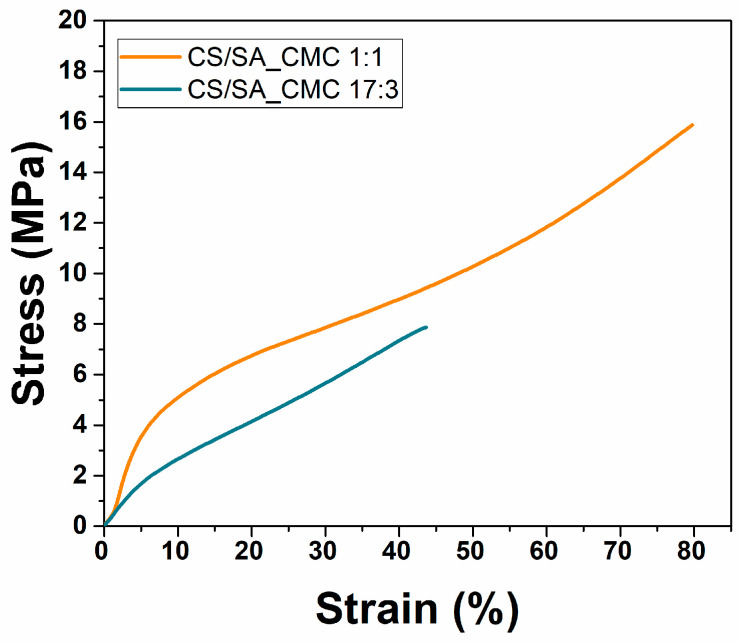
Mechanical properties of composite polysaccharide films. Stress-strain curves of chitosan/sodium alginate_sodium carboxymethyl cellulose films at a weight ratio of 1:1 (orange) or 17:3 (blue).

Mulching films must also resist external damage during storage and use, be flexible during application to the soil, and prevent matrix collapse [41,44,45]. Biodegradable and bio-based mulch films must retain similar mechanical properties as low-density polyethylene film, complying with standardized and international-specific norms, i.e., EN 17033 [46]. According to the latter, the biodegradable mulch films should have 16 MPa of tensile strength (σ) and 150% elongation at break (ε), which estimates the film’s toughness. An appropriate elongation is essential for avoiding breakage during film application on the soil. Films’ stress-strain curves and their related σ, ε, and elastic modulus (E) values (Figure 3 and Table 3) reinforce the importance of the polysaccharide ratio for their applicative properties.

The 1:1 film featured a σ value close to the required one (15.9 MPa), almost double that of the 17:3 counterpart (7.9 MPa). A similar trend was observed for ε, although its value was not comparable to that required for mulching films. Variations in the mechanical properties between the two films likely depend on the different networks, the extent of which was higher for the 1:1 film than the 17:3 film. This event increased the intermolecular forces and the tensile strength of the 1:1 film. In the CS/SA_CMC 17:3 film, the excess of chitosan likely alters the configuration of its polymer chain, potentially causing it to coil and exposing fewer hydrophilic sites to interact and form hydrogen bonds with SA and CMC. In turn, chitosan in excess might segregate on the surface, hindering the formation of a continuous polymer network and making the film less compact, resulting in the opposite outcome. Either way, CS molecules contain several amino and hydrogen groups, which form strong hydrogen bonds and prevent molecular slip, thereby reducing their elongation capability [3]. According to these considerations, we detected that the elastic modulus of the 1:1 film was much higher than that of the 17:3 sample. Thus, based on the obtained results, the 1:1 composite film could be the most promising for mulching applications.

#### 3.1.1. Effect of Adding NH_4_H_2_PO_4_ Salt

The biodegradable polysaccharide-based composite films were enriched with 30, 70, or 90% NH_4_H_2_PO_4_ (Section 2.4). The DS values of salt-enriched films decreased as the concentration of added NH_4_H_2_PO_4_ increased (Table 4), likely due to the elevated amount of salt compared to the total polymeric weight. A high salt content likely causes the polysaccharide chains to draw closer and interact, forming a tighter mesh network, aligning with the low degree of swelling. Considering the same salt concentration, the 1:1 films swelled more than their 17:3 counterparts, as observed in the above section.

**Figure 4:**
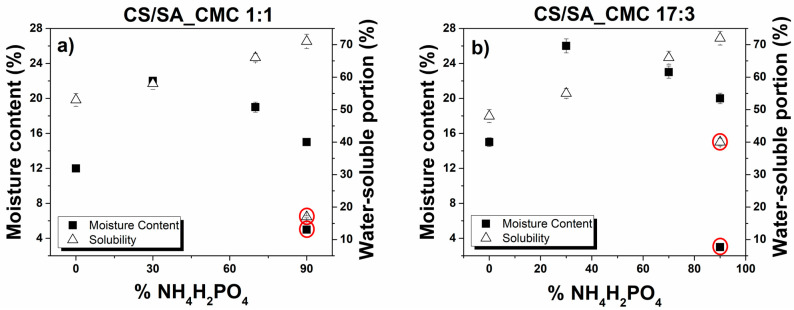
Water-interaction properties of composite polysaccharide films enriched with ammonium phosphate monobasic salt. Moisture content and water solubility as a function of NH_4_H_2_PO_4_ salt concentration of chitosan/sodium alginate_sodium carboxymethylcellulose in a weight ratio of (**a**) 1:1 or (**b**) 17:3 film. The red symbol (O) identifies the values of MC (%) and water-soluble portion (%) of crosslinked composite films.

The salt-enriched films also showed MC values higher than the reference films, yet an increasing salt content from 30 to 90% determined a continuous reduction of these values (Figure 4).

This outcome confirms our previous hypothesis, i.e., as the salt content increases, the polymer chains interact between them by forming more hydrogen bonds, reducing the number of polysaccharide hydrophilic sites available for interacting with water molecules. In line with these observations, the water-soluble portions of films enriched with NH_4_H_2_PO_4_ improved with increasing salt content, regardless of the film composition. Films enriched with 90% NH_4_H_2_PO_4_ showed the most promising water and moisture resistance and underwent further investigation. Yet, the salt dispersed within these films worsens their thermal and mechanical properties (Figures S3–S5; Tables S2 and S3). The presence of NH_4_H_2_PO_4_ between polymer chains caused a decrease in the maximum thermal degradation temperature from 248 °C to 215 °C (Figure S3a) and from 238 °C to 217 °C (Figure S4a) for the 1:1 and 17:3 films, respectively, inducing a change in their structural organization. The tensile strength property of the 1:1 film decreased from 15.8 MPa to 10.1 MPa, and the ε values varied from 79.7% to 16.2% (Figure S5a and Table S2), respectively. This phenomenon could be due to the inability of composite films containing a high content of NH_4_H_2_PO_4_ to form a compact and continuous matrix structure. The insertion of NH_4_H_2_PO_4_ into the polymer chains also increases the distance among adjacent chains, limiting the sliding and mobility between them and consequently decreasing the elongation at break. Accordingly, NH_4_H_2_PO_4_ enhanced the rigidity of the 1:1 film, as evidenced by the increased E value (from 88.8 to 359 MPa). However, the mechanical properties of salt-enriched 17:3 films did not change significantly. The σ value of the film decreased slightly from 7.9 MPa to 7.2 MPa, the ε value varied from 43.6% to 58.7%, and E changed from 35.9 to 59.9 MPa (Figure S5b and Table S3). In this case, NH_4_H_2_PO_4_ did not alter the cohesion force of the film, yet it likely acted as a filler, reinforcing the film network and enhancing its elongation at break [47].

#### 3.1.2. Effect of Adding CaCl_2_ Crosslinker

The negative impact of NH_4_H_2_PO_4_ on the films’ thermal and mechanical properties compelled us to identify a strategy to modify and improve these materials to enhance their suitability for mulching. Thus, the effect of a crosslinker, i.e., calcium chloride (CaCl_2_), was explored since crosslinking allows for obtaining dense polymer networks. Indeed, CaCl_2_ is known as a physical crosslinker that interacts with carboxylic groups of polysaccharides by forming the so-called egg-box structure [22,24]. Evidence regarding the physical crosslinking between Ca^2+^ and -COO^−^ ions of polysaccharides was first provided by the drastically decreased DS, water-soluble portion, and MC values of CaCl_2_-submerged salt-enriched films compared to their uncrosslinked counterparts (Figure 4 and Table 4). The crosslinking also increased the thermal stability of salt-enriched films and some strength characteristics. Indeed, the thermal degradation temperature and elastic modulus increased for the CaCl_2_-submerged 1:1 (252 °C, 1180 MPa) and 17:3 films (248 °C, 1531 MPa) compared to those of the uncrosslinked ones (Figures S3–S5; Tables S2 and S3). Similarly, the σ value for the Ca^2+^ crosslinked 17:3 film improved from 7.9 MPa to 36.2 MPa (Figure S5b and Table S3), whereas it remained constant for the 1:1 film (15.7 MPa; Figure S5a and Table S2). Conversely, the ε values dramatically decreased in the 1:1 (1.9%) and 17:3 (3.7%) crosslinked salt-enriched films (Tables S1 and S2). These results align with the literature regarding the physical crosslinking exerted by CaCl_2_ onto polysaccharides [22,24,25]. In this regard, the formation of the egg-box structure determines the disruption of the hydrogen bond network within films, the partial generation of strong polysaccharide-cations complexes, and a decreased availability of hydrophilic sites to bind with water [22,24,25]. In turn, these events decrease the spaces between polymeric chains, trap the salt into the polysaccharide matrix, hinder water penetration and absorption, generate a denser and semi-interpenetrating polymer network within the films, block the chains’ entanglement by partially immobilizing them, and reduce their free volume, conferring to the films a more compact and resistant structure. Yet, the crosslinking also reduces the flexibility of the polysaccharide matrix and increases its stiffness, making the films less deformable and reducing their elongation at break [22,24,25].

The results so far show that the polysaccharide ratio and the crosslinker presence significantly influence the macroscopical features of films, making these parameters crucial for optimizing such materials. In turn, this outcome makes it necessary to analyze the film structure to comprehend, at the molecular level, the cause-and-effect relationship between their composition and properties.

### 3.2. Structural Characterization of Polysaccharide-Based Composite Crosslinked Films

#### 3.2.1. ATR-FTIR Spectroscopy

**Figure 5:**
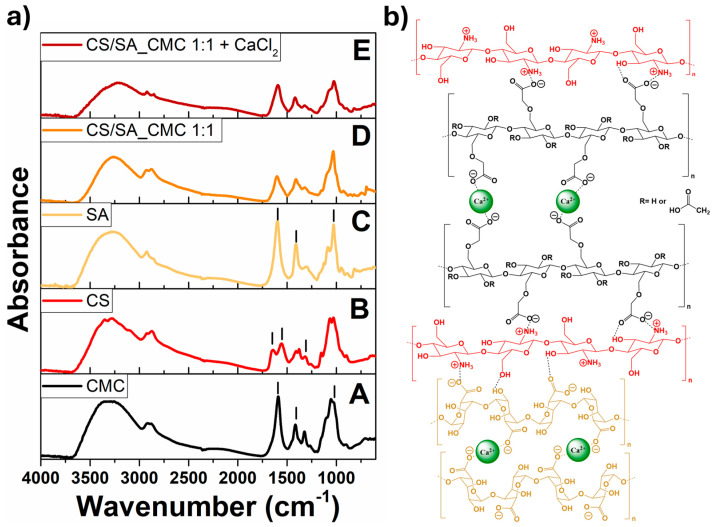
Structural characterization of crosslinked and uncrosslinked composite polysaccharide films. (**a**) ATR-FTIR spectra of (A) CMC, (B) CS, and (C) SA pure film and chitosan/sodium alginate_sodium carboxymethylcellulose film at a weight ratio of 1:1 (D) before and (E) after the addition of CaCl_2_. Symbol (**|**) indicates peaks of functional groups characteristic of each polysaccharide. (**b**) Schematic representation of interactions between CS, SA, and CMC, alongside those involving these polysaccharides and Ca^2+^ ions within the crosslinked films.

The ATR-FTIR spectra of the pure CS, SA, and CMC film and the CS/SA_CMC 1:1 composite film before and after CaCl_2_ treatment are shown in Figure 5a.

All spectra showed the characteristic bands at 3300 cm^−1^ and 2931 and 2862 cm^−1^ attributed to the -OH and the asymmetric and symmetric -CH_x_ stretching vibrations, respectively [48]. The spectra of the SA and CMC films also displayed bands typical of the asymmetric (ca. 1600 cm^−1^) and symmetric (ca. 1410 cm^−1^) -COO^−^ stretching vibrations [49,50] and a strong absorption band around 1023 cm^−1^ related to glycosidic linkages [51,52,53]. The CS film spectrum exhibited bands of -OH (3356 cm^−1^) and -NH (3282 cm^−1^, amide A) stretching vibrations [54,55,56], the amide I (1652 cm^−1^) and II (1590 cm^−1^) vibrational modes, and the C-O stretching vibration (1080 cm^−1^) [53,55,57].

**Figure 6:**
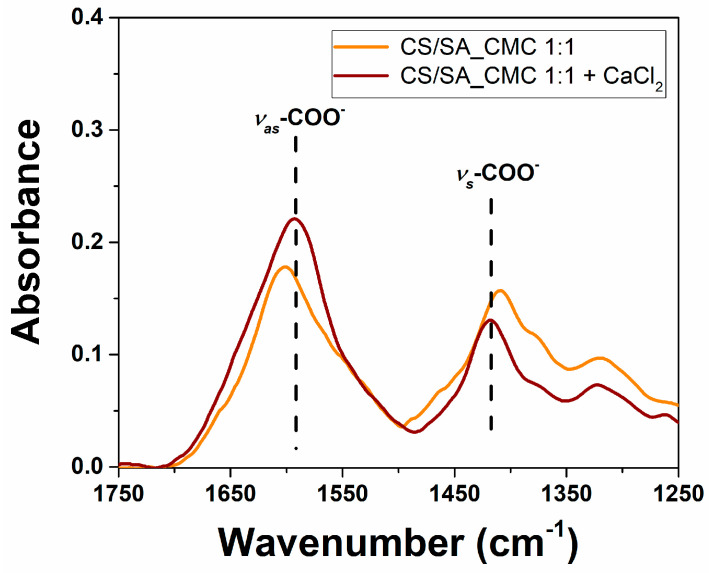
Structural modifications within composite polysaccharide films caused by crosslinking. Overlapping of the ATR-FTIR spectra of chitosan/sodium alginate_sodium carboxymethylcellulose films before and after the addition of CaCl_2_ in the spectral region from 1750 to 1250 cm^−1^.

In the CS/SA_CMC 1:1 composite film spectrum, the band at ca. 3200 cm^−1^ related to the -OH and -NH stretching vibrations was less intense, broader, and shifted to lower wavenumbers than pure compound films. This outcome may be due to the formation of more hydrogen bonds between CMC, CS, and SA that increase the chemical bond degree of polarization. Moreover, CaCl_2_ acted as a complexing agent for SA carboxylate anions, creating a new environment around the -COO^−^ group [50] by changing the charge density of the cation (from Na^+^ in the alginate blocks to Ca^2+^) [58]. Indeed, the CaCl_2_ crosslinking caused a significant redshift (from 1603 to 1594 cm^−1^) and blueshift (from 1409 to 1417 cm^−1^) of the -COO^−^ asymmetrical and symmetrical stretching vibrations, respectively (Figure 6) [58], evidencing the strong interaction between the polysaccharide -COO^−^ groups and Ca^2+^ ions [50].

Yet, the -COO^−^ bands in the composite film spectrum are typical of both SA and CMC and are superimposed; thus, the interaction of Ca^2+^ ions with the COO^−^ groups of CMC cannot be excluded. Overall, the more crosslinked polymeric structure resulting from the ionic interactions and hydrogen bonding within CaCl_2_-treated CS/SA_CMC 1:1 film diminishes the network space among polymeric chains and decreases the number of free –COO^−^ groups [59], limiting their interaction with water molecules, in turn justifying the film’s macroscopic properties. A schematic representation of interactions between different polysaccharides, alongside these substances and Ca^2+^ ions, is displayed in Figure 5b.

Although infrared spectroscopy confirmed the crosslinking events and supported our hypotheses regarding the structural changes in the composite film, it did not allow for discriminating the structural differences between the 1:1 and 17:3 films or investigating the effects of salt enrichment on their structure. Indeed, only vibrational modes typical of NH_4_H_2_PO_4_ were visible in the spectra of crosslinked or uncrosslinked films enriched with the salt (Figures S6–S8).

#### 3.2.2. XRD Analysis and Crystalline Structure of Composite Films

**Figure 7:**
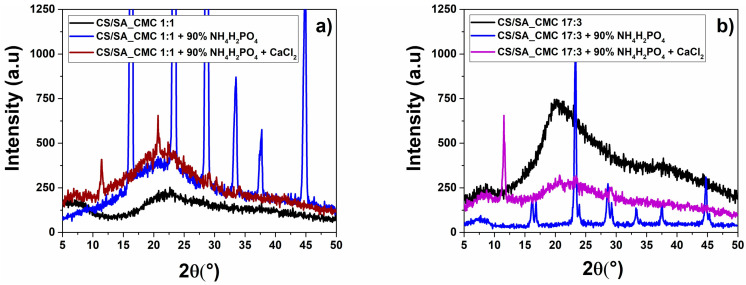
Crystalline structure of composite polysaccharide films, NH_4_H_2_PO_4_ enriched films, and/or crosslinked films. The XRD patterns of chitosan/sodium alginate_sodium carboxymethylcellulose films at weight ratio (**a**) 1:1 or (**b**) 17:3 film.

XRD analysis was performed to gain more information on the film structure changes induced by NH_4_H_2_PO_4_ and CaCl_2_. The XRD patterns of reference films presented broad diffraction peaks and a wide amorphous scattering halo (Figure 7), typical of polysaccharides with low crystallinity [60].

Nevertheless, the 17:3 film featured a more resolved peak at ca. 20° than the 1:1 sample, deriving from the CS/SA excess over CMC [61]. Both films enriched with NH_4_H_2_PO_4_ showed diffraction peaks at 16.4°, 16.8°, 23.4°, 23.9°, 28.6°, 29.2°, 33.5°, 37.7°, and 44.9°, which are characteristic of the salt. Conversely, the crosslinked NH_4_H_2_PO_4_-enriched films exhibited an overall more amorphous structure than the uncrosslinked ones [62], although a new peak at 2θ = 10.4° was detected and attributed to the (020) plane, representing the CS acetamido groups [63]. The salt-enriched crosslinked 1:1 film also showed a second peak at 2θ = 20.7° attributed to the (100) plane and assigned to the CS crystal lattice [63], which was absent for the 17:3 counterpart. This evidence suggests that the crosslinked films did not contain NH_4_H_2_PO_4_ in their crystal form; more likely, CaCl_2_ treatment causes modifications in the local organization of both polysaccharides and the NH_4_H_2_PO_4_ salt, which seems to be dispersed and entrapped in the polymeric matrix. CaCl_2_-induced film modifications indicated that the crosslinking event determined a certain order of degree in the polysaccharide structure and molecular chain entanglement [25], confirming the ATR-FTIR results. The peaks observed for the crosslinked films were attributable to the CS semi-crystalline domains of unplasticized CS, in agreement with TGA [64]. Thus, the coordination of -COO^−^ groups of SA and CMC with CaCl_2_, observed through FTIR spectroscopy, may have freed a portion of CS, in line with the TGA results. This CS excess became more ordered in the crosslinked than uncrosslinked films, as indicated by the more intense peak, representing acetamido groups that generate hydrogen bonds, allowing the easy integration of water to form hydrated crystals [63,65].

To further confirm these observations, the degree of crystallinity (DC; i.e., the percentage of crystalline regions over the total material) was calculated from XRD patterns (Table 5).

Uncrosslinked salt-enriched films featured the highest DC values, deriving from the NH_4_H_2_PO_4_ crystal deposits probably on the film surfaces. The crosslinked counterparts showed a lower DC than the latter, confirming the absence of salt crystals on the film and the crucial modifications caused by CaCl_2_ on its crystalline structure. In this regard, the crosslinker exerted opposite effects on the 1:1 and 17:3 films. CaCl_2_ slightly improved the 1:1 film crystallinity, whereas the 17:3 counterpart displayed an 11% decrease in the DC value. This outcome infers the importance of the polysaccharide amounts and ratios on the structural properties of films. Indeed, although the same coordination events between Ca^2+^ and -COO^−^ occur in both films, the 17:3 one contains an excess of CS/SA. The coordination event likely breaks apart the intermolecular hydrogen bond network between SA and CS, which are the majority, resulting in decreased film crystallinity. Moreover, since the crosslinking of CS generally produces more amorphous materials [61], the CaCl_2_ treatment likely hinders the formation of hydrogen bonds typically found in the CS semi-crystalline structures, which will be more abundant when the CS amount is high in the film. Finally, the absence of NH_4_H_2_PO_4_ crystals in the crosslinked films may infer a potential interaction between salt ions and polysaccharides that can contribute to the overall modification of the film’s crystalline structures and justify the changes in macroscopic properties observed.

### 3.3. Release Kinetics of PO_4_^3−^ and NH_4_^+^ Ions

**Figure 8:**
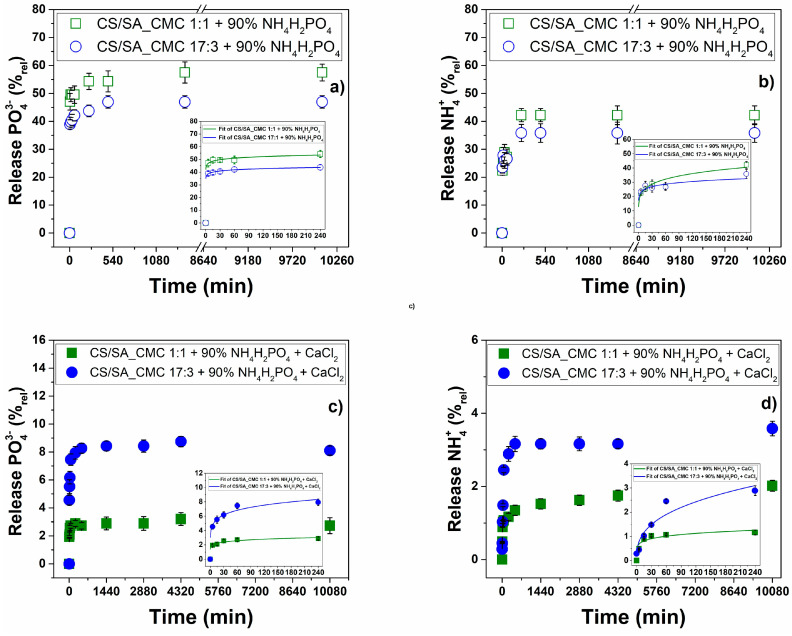
NH_4_H_2_PO_4_ release from composite polysaccharide films. The amount of PO_4_^3−^ and NH_4_^+^ ions released is reported as a relative percentage value as a function of time in (**a**,**b**) the absence or (**c**,**d**) the presence of CaCl_2_.

The kinetic of PO_4_^3−^ and NH_4_^+^ ions release over time was monitored for either crosslinked or uncrosslinked films to evaluate the effect of CaCl_2_ in this process, showing similar trends (Figure 8).

These trends are coherent with the rapid dissolution of the surface deposit of the excess salt. The CS/SA_CMC 1:1 film released the highest amount of PO_4_^3−^ and NH_4_^+^ ions per gram of film, implying salt trapping in the polysaccharide matrix. When the maximum swelling capacity is reached, the film network sufficiently expands to allow a fast salt diffusion of the polymer matrix [66]. From the DS results, the uncrosslinked salt-enriched 1:1 film swelled more than the 17:3 counterpart, indicating massive water penetration in the former, which agrees with the salt release results.

The release profiles of crosslinked films differed from those without CaCl_2_ (Figure 8). Indeed, the crosslinker minimizes the initial sudden release (bursting effect) shared by uncrosslinked matrices (Figure 8a,b) and slows the salt release rate. In this case, the CS/SA_CMC 17:3 films release the highest amount of salt. In line with the XRD analysis, a more amorphous structure determines a higher swelling capacity in water, allowing sufficient polysaccharide network expansion and leading to a faster diffusion of salt [66]. Finally, the quantity of NH_4_^+^ released is lower than that of the PO_4_^3−^ ion, supporting the hypothesis deduced from the XRD analysis of a possible interaction of salt with polymer chains.

The release data were processed through various kinetic models commonly reported in the literature for molecules within polymeric matrices [33]. The best model describing the mechanism of salt release was the Korsmeyer-Peppas model. This model is applicable when more than one type of analyte release phenomenon is involved [67]. In this regard, the analyte is released from hydrophilic matrices by diffusion through the polymeric matrix, its erosion (i.e., dissolution of the polymer), or a combination of the two [68]. These phenomena are due to the slow rearrangement of polymer chains and the diffusion process, which simultaneously cause time-dependent anomalous effects [69]. The release exponent (*n*) and correlation coefficient (R^2^) derived by fitting the experimental data (Figure 5) are shown in Table 6. 

The release exponent values ranged between 0 and 1, indicating a combination of Fickian and non-Fickian behaviors. The Fickian model (*n* = 0.5) considers the release dominated by diffusion, which generally occurs in polymeric matrices with highly mobile chains that enable easy solvent penetration. The non-Fickian or anomalous (*n* ≠ 0.5) model involves the release influenced not only by diffusion but also by swelling, relaxation, or other non-linear processes. The release exponent values mainly suggest a type of non-Fickian diffusion of salt. Specifically, the release exponent (*n*) is less than 0.5, ascribable to Sub-Fickian (or Case II Transport) diffusion. Therefore, the release process is slower than Fickian diffusion. Only for NH_4_^+^ ions released from the crosslinked film CS/SA_CMC 17:3, *n* > 0.5 ascribable to diffusion Super Fickian (or Case I Transport). In this case, the release process is faster than that predicted by Fick’s laws, likely due to additional mechanisms that facilitate the release of the salt to the solvent. This evidence is consistent with the XRD analysis (Table 5 and Figure 7) and the solubility test results (Figure 4). The 17:3 crosslinked film is structurally the most amorphous of all and more soluble than the 1:1 crosslinked film, indicating that the diffusion process is accompanied by the simultaneous erosion of the polymer matrix.

The results demonstrate that using films based on CMC, CS, and SA, regardless of their composition, PO_4_^3−^ ions are released within 30–60 min of the film’s contact with water, while 4 h (240 min) are needed for NH_4_^+^ ion release. The crosslinking of films with CaCl_2_ barely affects the ion release time but drastically reduces the total number of ions that can be released. These parameters are crucial for agricultural applications, as the quantity and timing of fertilizer release need to be carefully optimized depending on the crop type and environmental conditions. Although soil fertilization through mulching film degradation requires a slow and constant release of available nutrients, in the present study, we wanted to mimic extreme rainfall conditions or possible soil irrigation during plant growth, considering the general affinity of CS, SA, and CMC with water. Therefore, these formulations are good starting points for adding innovative and functional properties to mulching films and may represent a new approach to soil-sustainable fertilization.

## 4. Conclusions

In this study, sodium carboxymethyl cellulose, chitosan, and sodium alginate were combined at different weight ratios in the presence of glycerol as a plasticizer agent to obtain biodegradable composite films for agricultural purposes. The results demonstrated that combining polysaccharides in a weight ratio of 1:1 plays a fundamental role in optimizing the functional properties of the films, allowing for a more crosslinked structure and, consequently, a lower expanded network than using a 17:3 ratio. These films were also enriched with the inorganic salt NH_4_H_2_PO_4_ as a possible slow-release fertilizer, and the effect of CaCl_2_ as a crosslinker on some of their macroscopic properties and the release kinetics of NH_4_^+^ and PO_4_^3−^ ions were explored. The crosslinking efficiently reduced the film’s interaction with water, increased its thermal stability and strength characteristics, and slowed the rate of NH_4_^+^ and PO_4_^3−^ release. However, films did not feature the elongation at break required by EN 17033 for mulching applications. Therefore, although composite films based on natural polysaccharides and enriched with NH_4_H_2_PO_4_ as fertilizer can serve as innovative supports for agricultural mulches, further designing research activities for increasing and optimizing their elongation capacity is required to improve the suitability of films for their function.

## Figures and Tables

**Table 1:** Composition of the chitosan (CS), sodium alginate (SA), and carboxymethyl cellulose (CMC)-containing dispersions.

Dispersion Acronym	Description
CS/SA	1.5 g of CS and 1.5 g of SA with added 2 g of GLY (2.0% *w*/*v* to the total volume of the CS/SA dispersion) in 100 mL of a 2.0% *v*/*v* aqueous acetic acid solution.
CMC	1.5 g of CMC in 100 mL of water.
CS/SA_CMC 1:1	50 g of CS/SA dispersion and 50 g of CMC dispersion, weight ratio of 1:1.
CS/SA_CMC 17:3	85 g of CS/SA dispersion and 15 g of CMC dispersion, weight ratio of 17:3.
CS/SA_CMC 1:1 + 30% or 70% or 90% NH_4_H_2_PO_4_	Addition of 0.12 g, 0.28 g, and 0.36 g of NH_4_H_2_PO_4_ (30%, 70%, and 90%, respectively, than to total dry polymer mass, ~0.4 g) to the CS/SA_CMC dispersion in a weight ratio of 1:1.
CS/SA_CMC 17:3 + 30% or 70% or 90% NH_4_H_2_PO_4_	Addition of 0.12 g, 0.28 g, and 0.36 g of NH_4_H_2_PO_4_ (30%, 70%, and 90%, respectively than to total dry polymer mass, ~0.4 g) to the CS/SA_CMC dispersion in a weight ratio of 17:3.

**Table 2:** Main thermogravimetric events of the 1:1 and 17:3 composite films.

Samples	DTG_1_ (°C)	DTG_2_ (°C)	DTG_3_ (°C)	Weight Loss (%) at 200 °C	Residue (%) at 450 °C
CS/SA_CMC 1:1	71	160	248	17	32
CS/SA_CMC 17:3	71	175	238	23	26

DTG is the first derivative thermogravimetric and the subscripts 1, 2, and 3 are related to the first, second, and third thermal events.

**Table 3:** Mechanical Properties of the 1:1 and 17:3 composite films.

Samples	σ (MPa)	ε (%)	E (MPa)
CS/SA_CMC 1:1	15.9 ± 0.5	79.7 ± 2.5	88.8 ± 2.7
CS/SA_CMC 17:3	7.86 ± 0.25	43.7 ± 1.2	35.9 ± 1.1

σ, ε, and E represent tensile strength, elongation at break, and elastic modulus, respectively.

**Table 4:** Effect of adding NH_4_H_2_PO_4_ salt on the degree of swelling (%).

Samples	DS (%)				
NH_4_H_2_PO_4_ (%)	0	30	70	90	90 + CaCl_2_
CS/SA_CMC 1:1	1968 ± 39	524 ± 14	459 ± 13	325 ± 9	75 ± 2
CS/SA_CMC 17:3	1524 ± 31	286 ± 9	147 ± 4	134 ± 4	80 ± 2

**Table 5:** Degree of crystallinity (DC) of films.

Samples	DC (%)
CS/SA_CMC 1:1	20.8
CS/SA_CMC 1:1 + 90% NH_4_H_2_PO_4_	51.4
CS/SA_CMC 1:1 + 90% NH_4_H_2_PO_4_ + CaCl_2_	26.3
CS/SA_CMC 17:3	28.9
CS/SA_CMC 17:3 + 90% NH_4_H_2_PO_4_	50.3
CS/SA_CMC 17:3 + 90% NH_4_H_2_PO_4_+ CaCl_2_	17.2

**Table 6:** Release exponent (*n*) and determination coefficient (R^2^) of PO_4_^3−^ and NH_4_^+^ ions.

Samples	PO_4_^3−^		NH_4_^+^	
	*n*	R^2^	*n*	R^2^
CS/SA_CMC 1:1 + 90% NH_4_H_2_PO_4_	0.034 ± 0.007	0.92	0.169 ± 0.035	0.96
CS/SA_CMC 17:3 + 90% NH_4_H_2_PO_4_	0.032 ± 0.003	0.96	0.102 ± 0.030	0.89
CS/SA_CMC 1:1 + 90% NH_4_H_2_PO_4_ + CaCl_2_	0.129 ± 0.022	0.90	0.157 ± 0.050	0.83
CS/SA_CMC 17:3 + 90% NH_4_H_2_PO_4_ + CaCl_2_	0.146 ± 0.024	0.95	0.655 ± 0.085	0.88

## Data Availability

The raw/processed data required to reproduce these findings cannot be shared at this time as the data also forms part of an ongoing study.

## Supplementary Materials

The following supporting information can be downloaded at: https://www.mdpi.com/article/10.3390/polym16162298/s1, Figure S1: (a) 0.5% *w*/*v* CS/SA_CMC film in a weight ratio 1:1, (b) 0.5% *w*/*v* CS/SA_CMC film in a weight ratio 17:3, (c) 0.5% *w*/*v* CS/SA_CMC film in a weight ratio 34:6; (d) 1.0% *w*/*v* CS/SA_CMC film in a weight ratio 1:1, (e) 1.0% *w*/*v* CS/SA_CMC film in a weight ratio 17:3, (f) 1.0% *w*/*v* CS/SA_CMC film in a weight ratio 34:6; (g) 1.5% *w*/*v* CS/SA_CMC film in a weight ratio 1:1, (h) 1.5% *w*/*v* CS/SA_CMC film in a weight ratio 17:3, (i) 1.5% *w*/*v* CS/SA_CMC film in a weight ratio 34:6; (l) 2.0% *w*/*v* CS/SA_CMC film in a weight ratio 1:1, (m) 2.0% *w*/*v* CS/SA_CMC film in a weight ratio 17:3, Figure S2: Water-interaction properties of composite polysaccharide films. (a) Degree of swelling (%) and (b) and water-soluble portion (%) of 0.5% CS/SA_CMC films in a weight ratio 1:1, 17:3 and 34:6, Figure S3: TG curves and DTG curves of chitosan/sodium alginate_sodium carboxymethylcellulose film in weight ratio 1:1 (a) enriched with 90% NH_4_H_2_PO_4_ and (b) after addition of CaCl_2_, Figure S4: TG curves and DTG curves of chitosan/sodium alginate_sodium carboxymethylcellulose film in weight ratio 17:3 (a) enriched with 90% NH_4_H_2_PO_4_ and (b) after addition of CaCl_2_, Figure S5: Stress-strain curves of chitosan/sodium alginate_sodium carboxymethylcellulose films in weight ratio (a) 1:1 or (b) 17:3, Figure S6: ATR-FTIR spectra of chitosan/sodium alginate_sodium carboxymethylcellulose film in weight ratio 1:1 or 17:3, Figure S7: ATR-FTIR spectra of chitosan/sodium alginate_sodium carboxymethylcellulose crosslinked films in weight ratio 1:1 or 17:3, Figure S8: ATR-FTIR spectra of chitosan/sodium alginate_sodium carboxymethylcellulose crosslinked films in weight ratio 1:1 or 17:3 enriched with 90% NH_4_H_2_PO_4_. ν and δ indicate stretching and bending vibration modes, respectively; Table S1: Main thermogravimetric events of the 0.5% CS/SA_CMC composite films in a weight ratio 1:1, Table S2: Mechanical properties of CS/SA_CMC 1:1 composite films, Table S3: Mechanical properties of CS/SA_CMC 17:3 composite films.
