# Supplementary material for: Exploring the Feasibility of Polysaccharide-Based Mulch Films with Controlled Ammonium and Phosphate Ions Release for Sustainable Agriculture
Source: Polymers (Basel). 2024 Aug 14;16(16):2298. doi: 10.3390/polym16162298 (PMC11359579; doi:10.3390/polym16162298)
Supplement: Supplementary file 1 [file polymers-16-02298-s001.zip › polymers-3082703-supplementary.pdf]

polymers

Supplementary materials for

Exploring the feasibility of polysaccharide-based mulch films with controlled ammonium and phosphate ions release for sustainable agriculture

Veronica Ciaramitaro [1], Elena Piacenza [1,*], Sara Paliaga [2], Giuseppe Cavallaro [3], Luigi Badalucco [2], Vito Armando Laudicina [2,*] and Delia Francesca Chillura Martino [1]

[1] Department of Biological, Chemical, and Pharmaceutical Sciences and Technology (STEBICEF), Università degli Studi di Palermo, Viale delle Scienze building 17, 90128, Palermo (Italy); veronicaconcetta.ciaramitaro@unipa.it elena.piacenza@unipa.it delia.chilluramartino@unipa.it

[2] Department of Agricultural, Food and Forest Sciences, Università degli Studi di Palermo, Viale delle Scienze building 4, Palermo (Italy); sara.paliaga@unipa.it luigi.badalucco@unipa.it vitoarmando.laudicina@unipa.it

[3] Department of Physics and Chemistry - Emilio Segrè, Università degli Studi di Palermo, Viale delle Scienze building 17, Palermo (Italy); giuseppe.cavallaro@unipa.it

* Correspondence: elena.piacenza@unipa.it; vitoarmando.laudicina@unipa.it

Preliminary optimization of polysaccharide-based films

Besides conditions described in the 2.2 and 2.3 subsections of the main manuscript, CMC dispersions were also prepared by dissolving 0.5, 1.0, and 2.0 g in 100 ml of distilled water, and CS/SA dispersions by dissolving 0.5, 1.0, and 2.0 g of CS and SA, respectively, in 100 ml of a 2% v/v aqueous acetic acid solution. To the latter dispersion, 2% w/v glycerol was added.

0,5%, 1.0%, and 2.0% (w/v) CS/SA and CMC dispersions were mixed at a CS/SA and CMC weight ratio of 1:1 and 17:3. To obtain thicker films, 34 g of the CS/SA dispersion was mixed with 6 g of the CMC dispersion. Aliquots of 10 g dispersions were cast on Petri dishes, dried in an oven for 24 h at 60 °C, and cooled at room temperature to obtain the films.

The films will be indicated as 0.5%, 1.0%, 1.5%, or 2.0% CS/SA_CMC 1:1, 17:3, or 34:6, depending on the polymer concentration and amount of each dispersion used to prepare the composite film.

Figure S1 shows the obtained films.

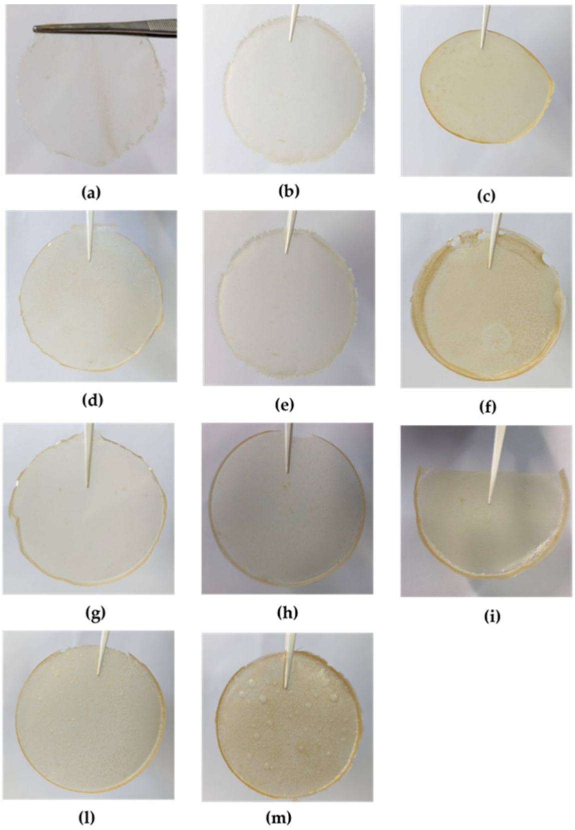

Figure S1. (a) 0.5% w/v CS/SA_CMC film in a weight ratio 1:1, (b) 0.5% w/v CS/SA_CMC film in a weight ratio 17:3, (c) 0.5% w/v CS/SA_CMC film in a weight ratio 34:6; (d) 1.0% w/v CS/SA_CMC film in a weight ratio 1:1, (e) 1.0% w/v CS/SA_CMC film in a weight ratio 17:3, (f) 1.0% w/v CS/SA_CMC film in a weight ratio 34:6; (g) 1.5% w/v CS/SA_CMC film in a weight ratio 1:1, (h) 1.5% w/v CS/SA_CMC film in a weight ratio 17:3, (i) 1.5% w/v CS/SA_CMC film in a weight ratio 34:6; (l) 2.0% w/v CS/SA_CMC film in a weight ratio 1:1, (m) 2.0% w/v CS/SA_CMC film in a weight ratio 17:3.

Figure S1 lacks the 2.0% CS/SA_CMC 34:6 film picture as it was impossible to mix the dispersions of CS/SA and CMC to obtain a liquid homogeneous dispersion to pour into Petri dishes. The macroscopically more homogeneous films were those whose CS/SA_CMC weight ratio was 1:1 and 17:3, obtained by mixing the CS/SA and CMC dispersions at a polymer concentration of 0.5 w/v % and 1.5 w/v%. Other compositions were discarded because the resulting films were inhomogeneous and rougher to the touch due to the formation of air bubbles, as can be observed from the images reported in Figure S1.

Before proceeding with deep analysis to understand the structure and interaction exerted among components in the films, we explored macroscopic properties such as the resistance to temperature, degree of swelling, and solubility of films obtained with a polymer concentration of 0.5% w/v.

The thermogravimetric data of the 0.5% CS/SA_CMC composite films are reported in Table S1, while the degree of swelling (DS) and water-soluble portion are shown in Figure S2.

Table S1. Main thermogravimetric events of the 0.5% CS/SA_CMC composite films in a weight ratio 1:1, 17:3 and 34:6.

Samples	DTG_1 (°C)	DTG_2 (°C)	DTG_3 (°C)	Residue (%) at 450°C
0.5% CS/SA_CMC 1:1	56	170	225	19
0.5% CS/SA_CMC 17:3	62	189	248	13
0.5% CS/SA_CMC 34:6	73	203	248	26

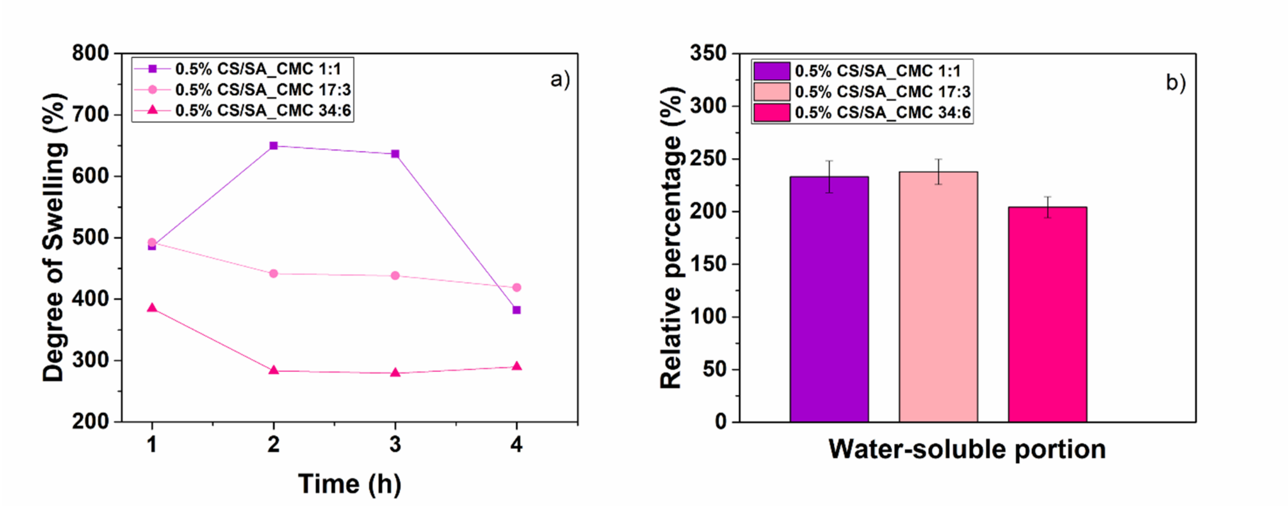

Figure S2. Water-interaction properties of 0.5% w/v polysaccharide films. (a) Degree of swelling (%) and (b) and water-soluble portion (%) of 0.5% CS/SA_CMC films in a weight ratio 1:1, 17:3 and 34:6.

For the 0.5% CS/SA_CMC films, the temperature corresponding to the maximum thermal decomposition rate was lower than that of the 1.5% CS/SA_CMC films (Figure 2 and Table 2), likely due to more crosslinking events between the CS, SA, and CMC molecules in the latter, which makes the film more compact.

Moreover, the 1.5% CS/SA_CMC films show a significantly higher water absorption (Figure 1) than 0.5% CS/SA_CMC films (Figure S2). Yet, the 0.5% CS/SA_CMC films dissolve in water after a short time, showing a higher loss in weight (ca. 250%) and lower water resistance than the 1.5% films, explaining their decrease of DS after 3h-soaking. The results suggest that in the presence of a higher CS and SA content, the polymer matrix hinders the permeation or the diffusion of water inside it, making it more resistant and more thermally stable.

Based on this evidence, the 1.5% CS/SA_CMC films were selected for deeper analysis, which is reported and discussed in the manuscript.

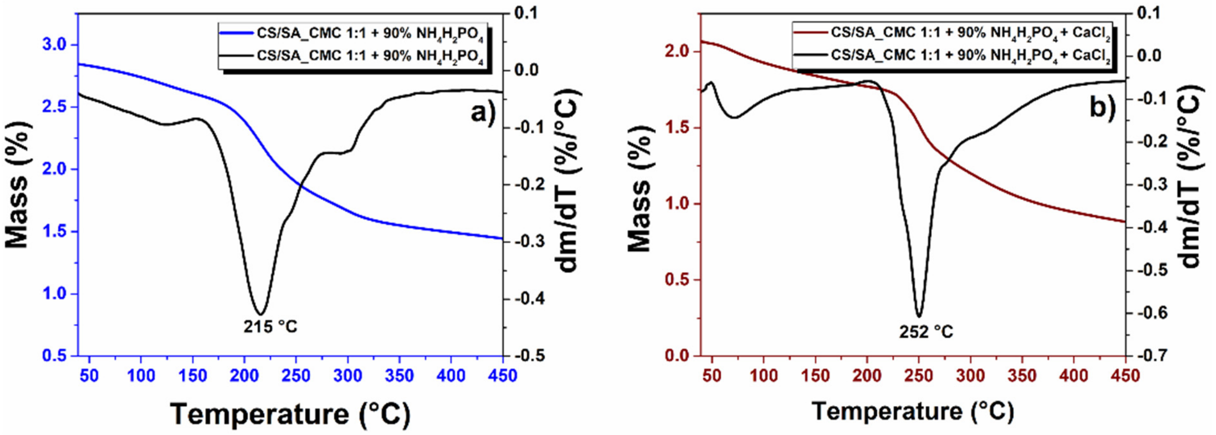

Figure S3. TG curves and DTG curves of chitosan/sodium alginate_sodium carboxymethylcellulose film in weight ratio 1:1 a) enriched with 90% of $NH_4H_2PO_4$ and b) after addition of $CaCl_2$.

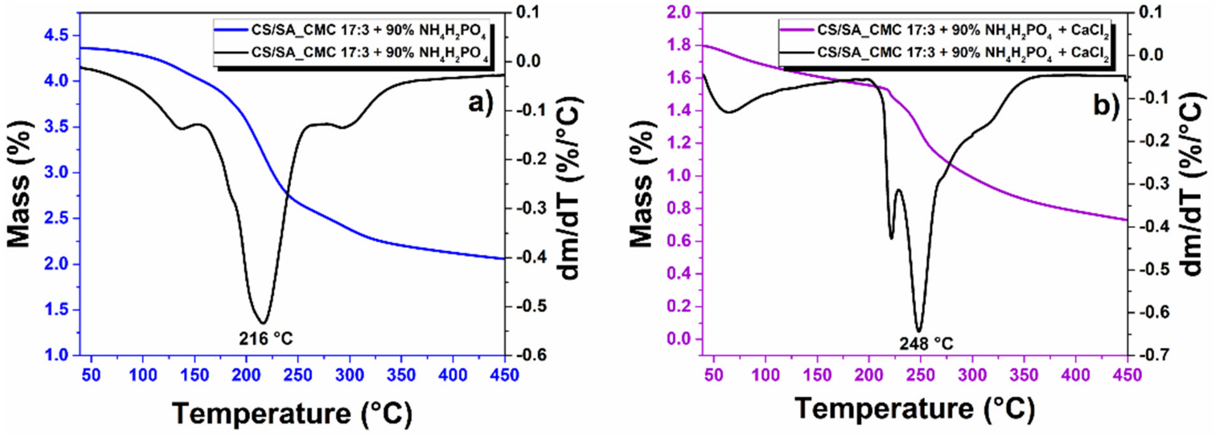

Figure S4. TG curves and DTG curves of chitosan/sodium alginate_sodium carboxymethylcellulose film in weight ratio 17:3 a) enriched with 90% of $NH_4H_2PO_4$ and b) after addition of $CaCl_2$.

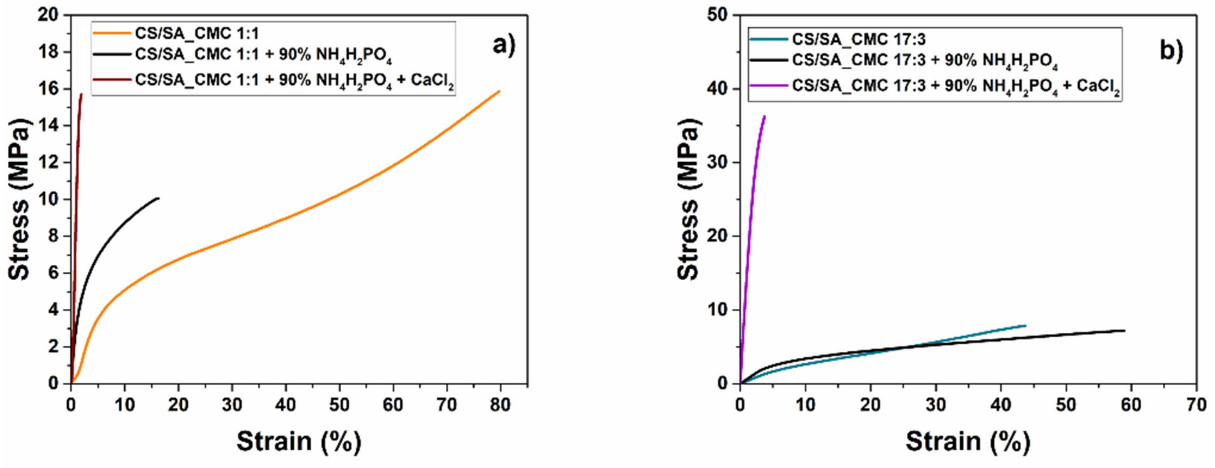

Figure S5. Stress-strain curves of chitosan/sodium alginate_sodium carboxymethylcellulose films in weight ratio a) 1:1 or b) 17:3.

Table S2. Mechanical properties of CS/SA_CMC 1:1 composite films.

Samples	σ (MPa)	ε (%)	E (MPa)
CS/SA_CMC 1:1	15.9 ± 0.5	79.7 ± 2.5	88.8 ± 2.7
CS/SA_CMC 1:1 + 90% $NH_4H_2PO_4$	10.1 ± 0.3	16.2 ± 0.5	358.9 ± 10.9
CS/SA_CMC 1:1 + 90% $NH_4H_2PO_4$ + $CaCl_2$	15.7 ± 0.5	1.92 ± 0.06	1180 ± 37

σ, ε, and E represent tensile strength, elongation at break, and elastic modulus, respectively.

Table S3. Mechanical properties of CS/SA_CMC 17:3 composite films.

Samples	σ (MPa)	ε (%)	E (MPa)
CS/SA_CMC 17:3	7.86 ± 0.25	43.7 ± 1.2	35.9 ± 1.1
CS/SA_CMC 17:3 + 90% $NH_4H_2PO_4$	7.21 ± 0.22	58.7 ± 1.8	59.9 ± 1.8
CS/SA_CMC 17:3 + 90% $NH_4H_2PO_4$ + $CaCl_2$	36.2 ± 1.1	3.71 ± 0.11	1531 ± 46

σ, ε, and E represent tensile strength, elongation at break, and elastic modulus, respectively.

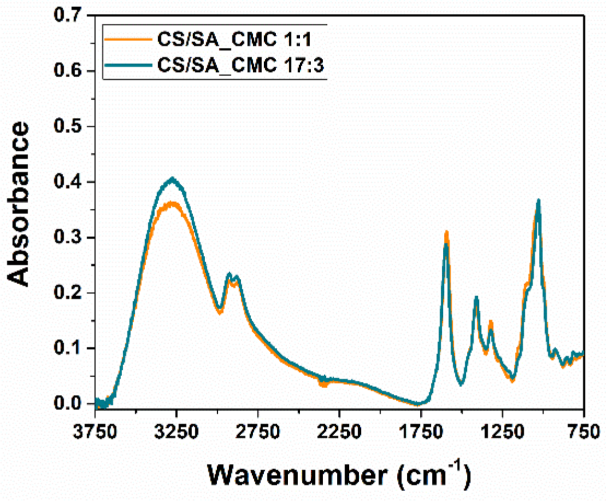

Figure S6. ATR-FTIR spectra of chitosan/sodium alginate_sodium carboxymethylcellulose film in weight ratio 1:1 or 17:3.

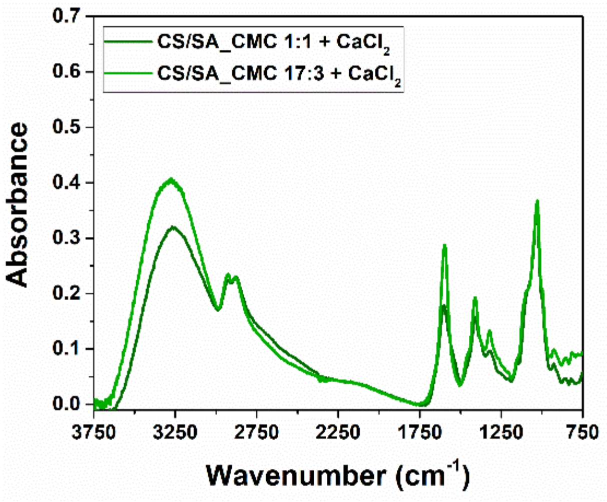

Figure S7. ATR-FTIR spectra of chitosan/sodium alginate_sodium carboxymethylcellulose crosslinked films in weight ratio 1:1 or 17:3.

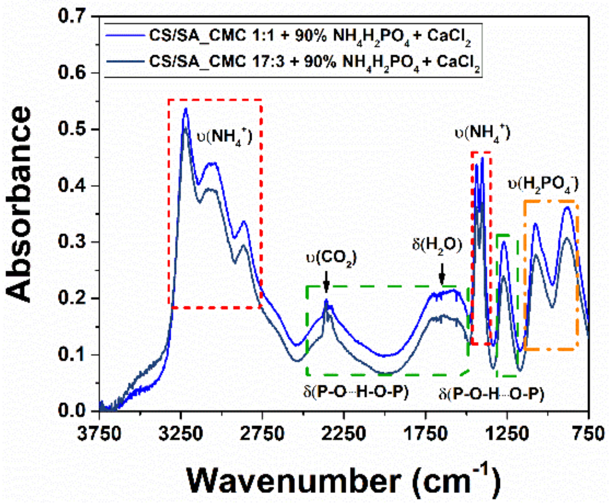

Figure S8. ATR-FTIR spectra of chitosan/sodium alginate_sodium carboxymethylcellulose crosslinked films in weight ratio 1:1 or 17:3 enriched with 90% of $NH_4H_2PO_4$. ν and δ indicate stretching and bending vibration modes, respectively.
